# Diet-induced changes in metabolism influence immune response and viral shedding in Jamaican fruit bats

**DOI:** 10.1098/rspb.2024.2482

**Published:** 2025-02-19

**Authors:** Caylee A. Falvo, Daniel E. Crowley, Evelyn Benson, Monica N. Hall, Benjamin Schwarz, Eric Bohrnsen, Manuel Ruiz-Aravena, Madison Hebner, Wenjun Ma, Tony Schountz, Agnieszka Rynda-Apple, Raina K. Plowright

**Affiliations:** ^1^ Department of Public and Ecosystem Health, College of Veterinary Medicine, Cornell University, Ithaca, NY 14853, USA; ^2^ Department of Microbiology and Cell Biology, Montana State University, Bozeman, MT 59717, USA; ^3^ Research and Technologies Branch, National Institute of Allergy and Infectious Diseases, National Institutes of Health, Hamilton, MT 59840, USA; ^4^ Department of Wildlife, Fisheries and Aquaculture, Mississippi State University, Starkville, MS 39762, USA; ^5^ Department of Veterinary Pathobiology, College of Veterinary Medicine and Department of Molecular Microbiology and Immunology, School of Medicine, University of Missouri, Columbia, MO 65211, USA; ^6^ Center for Vector-Borne Infectious Diseases, Department of Microbiology, Immunology, and Pathology, Colorado State University, Fort Collins, CO 80523, USA

**Keywords:** metabolomics, viral shedding, experimental infection, Jamaican fruit bats, nutrition

## Abstract

Land-use change may drive viral spillover from bats into humans, partly through dietary shifts caused by decreased availability of native foods and increased availability of cultivated foods. We experimentally manipulated diets of Jamaican fruit bats to investigate whether diet influences viral shedding. To reflect dietary changes experienced by wild bats during periods of nutritional stress, Jamaican fruit bats were fed either a standard diet or a putative suboptimal diet, which was deprived of protein (suboptimal-sugar diet) and/or supplemented with fat (suboptimal-fat diet). Upon H18N11 influenza A-virus infection, bats fed on the suboptimal-sugar diet shed the most viral RNA for the longest period, but bats fed the suboptimal-fat diet shed the least viral RNA for the shortest period. Bats on both suboptimal diets ate more food than the standard diet, suggesting nutritional changes may alter foraging behaviour. This study serves as an initial step in understanding whether and how dietary shifts may influence viral dynamics in bats, which alters the risk of spillover to humans.

## 1. Introduction

Zoonotic spillovers may be occurring more frequently, with anthropogenic land-use change cited as a major driver [1,2]. Land-use change impacts wildlife habitat structure, resource availability and animal behaviour [1,3]. Changing these ecological conditions can increase risk of spillover events, both through increasing contact rates between humans and wildlife and by impacting the immune systems of wildlife reservoir hosts [4]. Although the direct mechanisms linking land-use change to viral spillover are multivariate, changes in food availability leading to dietary shifts or nutritional stress are likely to play an important role.

Food availability, dietary shifts and nutritional stress can impact the immune response, which must compete with other physiological processes when food and energetic resources are limited [5]. Nutritional stress, such as caloric deficiency or imbalance, diverts resources from the immune system [6] and increases susceptibility to a variety of infections [7–9]. Studies in animal models have shown that low-protein diets increase the likelihood of viral shedding [10,11], increase disease severity [12] and reduce survival [8,12]. High-fat diets can increase infection severity and duration of viral shedding [13]. Although diet composition has been shown to impact viral infection in animal models, this has not been established in wildlife.

For many wild animals, habitat alteration has caused changes in behaviour, including foraging on anthropogenic food sources [14–21], which affects their health and immune status [22–24]. This influences levels of infection in populations [18,24,25], overall pathogen dynamics [18,26] and contact rates with other animals [18,27] and humans [15,20,24,28,29]. Despite limited research on the influence of diet on viral shedding in wild animals, increasing human–wildlife contact suggests it is important to characterize this effect to understand the health of wildlife and the potential for pathogen spillover.

Changes in foraging behaviour have been documented in multiple species of wild bats, including reservoir hosts of known and potential zoonotic viruses [15–17,29]. Disruption of native habitat causes bats to forage in human-modified landscapes where there are reliable cultivated food sources [15,16,29–32]. For example, Pallas’ long-tongued bats (*Glossophaga soricina*) in banana plantations switched to consuming banana nectar [31]. Egyptian fruit bats (*Rousettus aegyptiacus*) predominantly foraged in human-residential areas on cultivated fruits such as bananas, but moved back to natural areas as soon as native food was available there [15]. Flying foxes (*Pteropus* spp.) feed on cultivated plants (e.g. oranges, cocos palm) in anthropogenic landscapes owing to loss of native habitat [16,33,34], associated with increased Hendra virus shedding [35] and spillovers into horses [14]. The nutritional composition of cultivated foods tends to differ from native plants, often having lower protein and/or higher lipid content [23,36,37]. For example, frugivorous bats consume commercially grown bananas [31,32], which provide nectar but negligible pollen [38]—an important source of protein. Giant fruit-eating bats (*Artibeus lituratus*) [39] and black flying foxes (*Pteropus alecto*) [16] in urban areas consume non-native cocos palm (*Syagrus romanzoffiana*), which has substantially higher lipid content than most native fruits (approx. 20% lipid) [37]. Variation in dietary protein and lipid influences viral shedding patterns experimentally in model species [10,11,13]. Thus, in these systems, poor nutrition could increase viral shedding rates and ultimately increase the risk of spillovers.

To directly test the effects of dietary changes on viral shedding in bats, we infected Jamaican fruit bats (*Artibeus jamaicensis*; JFBs) with a virus that *Artibeus* bats naturally host, H18N11-influenza A virus (H18N11-IAV). JFBs are a tractable experimental bat species because they are small, breed well in captivity, and H18N11-IAV can be used in ABSL−2 (animal biosafety level 2) conditions. We assigned naive JFBs to diets that we believe represent nutritional limitations associated with dietary shifts in bats that are reservoirs of known or potential zoonotic viruses (e.g. *Artibeus spp*., *R. aegyptiacus*, *Eidolon helvum, Pteropus alecto*) [14,15,40]: a standard diet (fruit with protein supplement, similar to fruit and proteinaceous pollen) and two suboptimal diets (high sugar but low protein such as oranges; or high fat but low protein such as cocos palm). The JFBs were then infected with H18N11-IAV to address whether and how the suboptimal diets could increase viral shedding by inducing changes in metabolism and the immune response. We analysed the metabolome of the bats and several immune markers to provide a broader understanding of the influence of diet on their physiological status and how it responds to infection.

## 2. Results

### (a) Interaction between infection and diet influences food consumption

**Figure 1.**
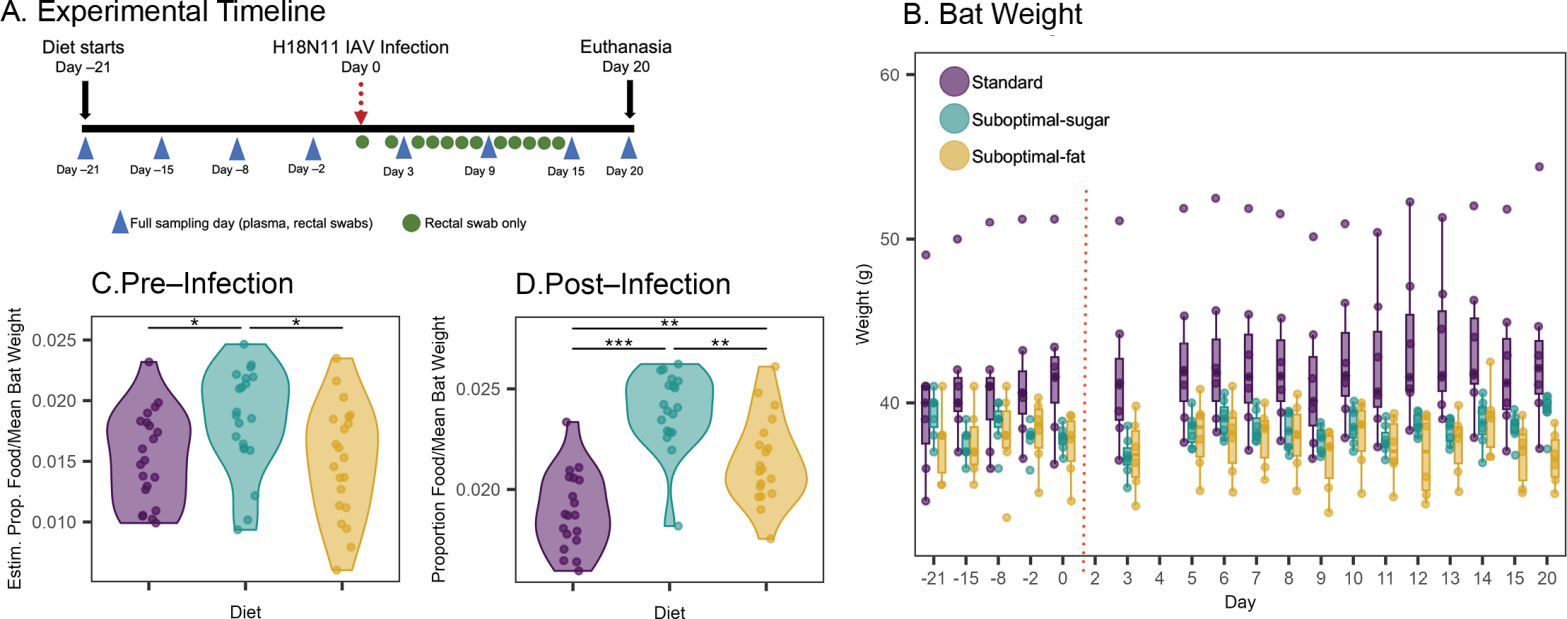
(A) Schematic of experimental timeline. (B) Weight of bats throughout the experiment. Standard diet bats were heavier on average, but between diet start and infection (day −21 and day 0) weight did not significantly vary. Post-infection (day 0 to day 20), standard and suboptimal-sugar diet bats gained a small amount of weight (linear mixed effects model output (lme), 0.06 g day^–1^ estimate; df = 257, *t* = − 3.92, *p* = 0.001), while suboptimal-fat diet bats lost a small amount of weight (lme, −0.02 g day^–1^; df = 257, t = −3.39, *p* = 0.008). Vertical dotted red line separates pre- versus post-infection days. (C) Pre-infection overall differences in estimated proportion of food consumed, based on average food provided minus weight of food leftover, corrected by average weight of bats in the cage. Suboptimal-sugar diet cages consumed more food than suboptimal-fat and standard cages (ANOVA, *F* = 4.32, df = 2, *p* = 0.02). (D) Post-infection overall food consumed, based on actual weight of food provided minus weight of food left over, corrected by average weight of bats in the cage. Suboptimal-diet cages consumed more food overall than standard diet bats, although suboptimal-sugar bats still consumed the most (ANOVA, *F* = 30.36, df = 2, *p* < 0.001). Boxplots show median, 25th and 75th percentiles and range or 1.5 × IQR. Asterisks indicate: *** *p* < 0.0005, ** *p* < 0.005, * *p* < 0.05, **·** *p* < 0.1.

The JFBs’ weights did not change from the beginning of the experiment until immediately pre-infection (figure 1A) in any of the diet groups (figure 1B). Post-infection, suboptimal-sugar and standard diet bats gained a small amount of weight (0.06 g day^–1^ average), while suboptimal-fat diet bats lost a small amount of weight (−0.02 g day^–1^ average). Despite the random selection of bats for each diet group, weights varied by treatment and the standard diet bats were slightly heavier on average (ANOVA, df = 2, *F* = 5.289, *p* = 0.013).

We measured food consumption to determine changes in appetite. Pre-infection, the suboptimal-sugar diet cage consumed the most food (figure 1C). Post-infection, both suboptimal diet cages consumed more food overall than standard diet bats, although suboptimal-sugar bats still consumed the most (figure 1D). Normalizing by number of bats per cage did not substantially change the results (electronic supplementary material, figure S1). Aside from the change in food consumption, the infection did not result in other clinical symptoms in bats.

### (b) Diet led to unique metabolomic profiles in rectal environment, plasma and liver

**Figure 2.**
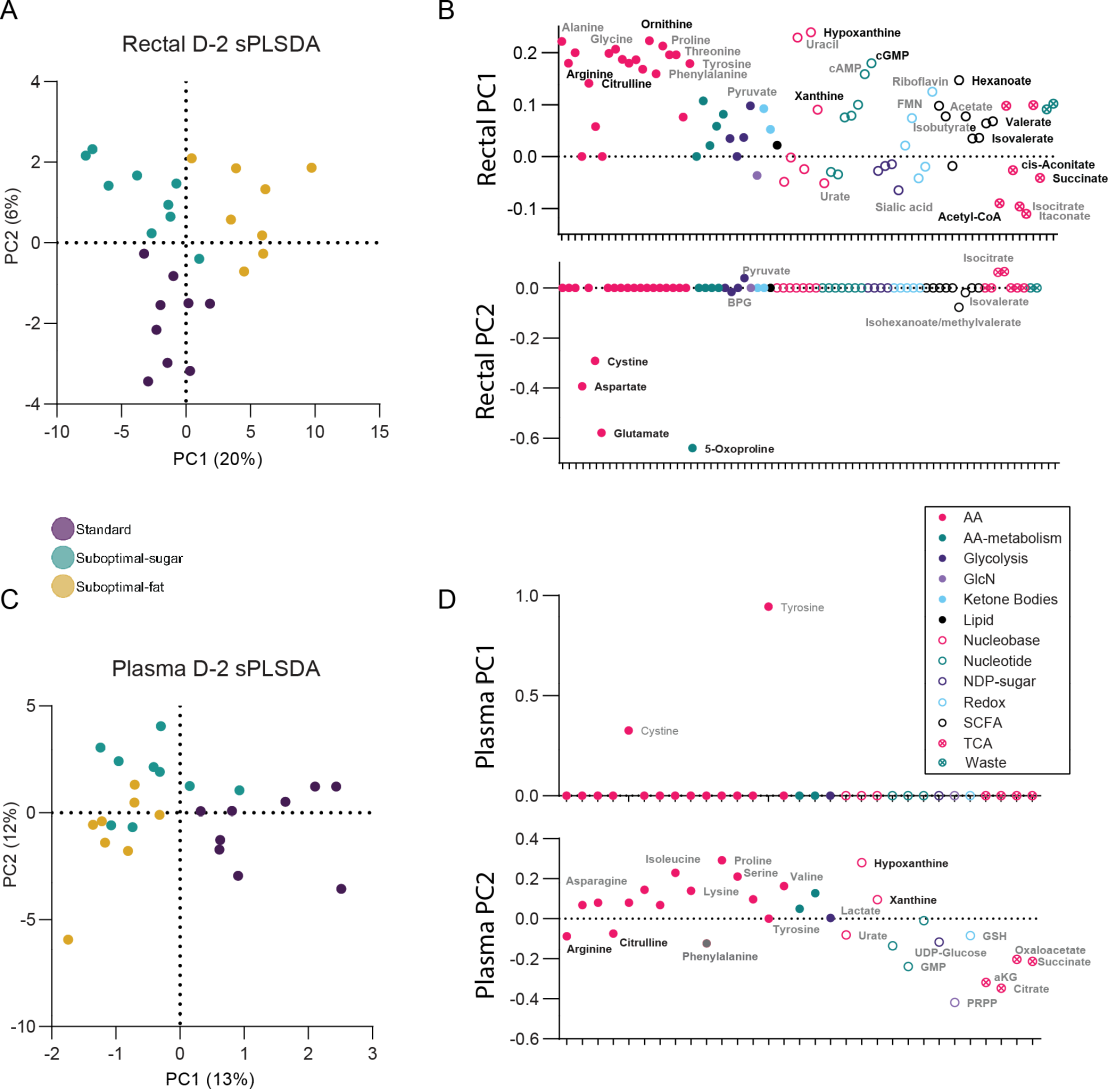
(A,C) Sparse partial least squares regression (sPLSDA) classification of bats in each diet pre-infection (day −2, D−2) based on metabolites in rectal swabs (A) and plasma (C). Each point represents an individual bat. (B,D) Loadings of metabolites from first two axes of the sPLSDA (PC1, PC2) for rectal swabs (B) and plasma (D). Each point represents a metabolite (shape- and colour-coded per legend). AA, amino acids; SCFAs, short-chain fatty acids; TCA, tricarboxylic acid cycle ; FMN, flavin mononucleotide; BPG, bisphosphoglycerate; NDP-sugar, nucleoside diphosphate-sugars; GlcN, glucosamine

We analysed rectal swabs, plasma and liver samples to determine how diet affected systemic and local metabolic profiles. We found that manipulating diet led to distinct metabolic profiles pre-infection in both the rectal environment and plasma (sparse partial least squares regression (sPLSDA); figure 2A,C) (liver only analysed at final/terminal time point). On average, differences were significant in the rectal compartment (PERMANOVA, *F* = 2.472, *p* = 0.03) but not in plasma (PERMANOVA, F = 1.773, *p* = 0.281). These metabolic groupings appeared stable by the point of infection and were largely maintained between diets post-infection (electronic supplementary material, figure S2).

In rectal swabs pre-infection (day −2), the suboptimal-fat diet was distinct from the other diets based on PC1 (Rectal PC1, figure 2A), whereas both suboptimal diets were distinguishable from standard diet based on PC2 (Rectal PC2, figure 2A). Divergence between diets was partly driven by differences in short-chain fatty acids (SCFAs), amino acids, tricarboxylic acid cycle (TCA) metabolites, basic sugars and nucleobases. Suboptimal-fat diet bats had decreased levels of several TCA-related metabolites (e.g. acetyl-CoA, succinate, isocitrate, *cis*-aconitate), suggesting different prioritization of energy metabolism in the host and/or microbial compartment (Rectal PC1, figure 2B). Suboptimal-fat diet bats had increased levels of most amino acids, SCFAs and nucleobases/nucleosides (e.g. cGMP, xanthine, hypoxanthine), suggesting increased production or turnover of protein and nucleobases/nucleosides. A subset of these metabolites (arginine, citrulline, hypoxanthine, several SCFAs) were notable, as they correlate positively with gut health and immune function in mice and humans. Both suboptimal diets had decreased levels of 5-oxoproline, glutamate, aspartate and cystine (Rectal PC2, figure 2B; electronic supplementary material, figure S2A). Surprisingly, the availability of most amino acids (except glutamate, aspartate and cystine) was highest in suboptimal-fat diet bats but lowest in suboptimal-sugar diet bats, despite both suboptimal diets being protein-poor (figure 2B).

In plasma, suboptimal diets clustered together but were distinguishable from standard diets (Plasma PC1, figure 2C). The suboptimal diets had lower levels of several amino acids compared with standard diet. This pattern contrasts with the elevated amino acids in the rectal environment of suboptimal-fat diet bats. Several nucleobases (hypoxanthine, xanthine) were lowest in the plasma of suboptimal-fat diet bats (electronic supplementary material, figure S2B), despite being elevated in their rectal environment (hypoxanthine; electronic supplementary material, figure S2A). The opposing patterns of amino acids and hypoxanthine between the rectal and plasma metabolome in the suboptimal-fat diet, but not in the suboptimal-sugar diet, suggests there are diet-driven differences related to the transport, synthesis or recycling of these base nutrients between host and microbes in the gut compartment. The suboptimal diets were not distinguishable in plasma (figure 2C), except for an elevation of citrulline and arginine in the suboptimal-fat bats relative to the suboptimal-sugar bats (Plasma PC2, figure 2D).

Diet-dependent patterns were easily discernible in the liver and reflected the main source of energy that the bats received (electronic supplementary material, figure 3A,B). Like plasma, both suboptimal diets were amino acid-depleted in the liver as expected from a protein-poor diet (electronic supplementary material, figure S3C). The notable exception was arginine, which was elevated in both suboptimal diets compared with the standard diet. Patterns consistent with higher sugar-based metabolism were apparent in both suboptimal diets, which was expected for the suboptimal-sugar diet but surprising for the suboptimal-fat diet. However, this pattern could also indicate higher levels of gluconeogenesis in the suboptimal-fat bats (as opposed to glycolysis), as these data cannot determine directionality for bidirectional pathways. As expected, levels of acetyl-CoA were highest in the suboptimal-fat diet bats, which suggests elevated fatty acid oxidation. Higher levels of ketone bodies (3-hydroxybutyrate, acetoacetate) in the standard compared with the suboptimal diets (electronic supplementary material, figure S3B) suggest that bats may use ketogenic metabolism to a higher degree when consuming a protein-supplemented diet.

**Figure 3.**
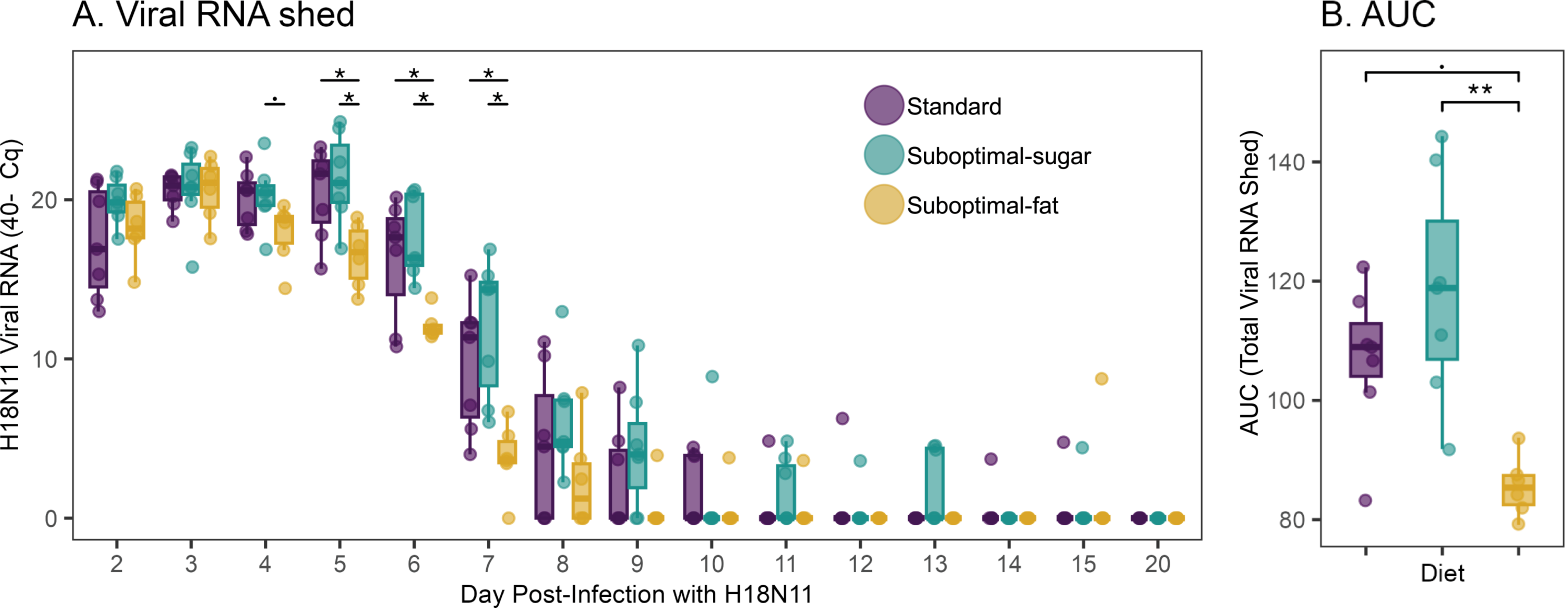
(A) Viral RNA detected post-infection (40-Cq). Viral RNA detected differed among diets on days 4−7 (ANOVA; day 4 *p* = 0.08, *F* = 2.911; day 5 *p* = 0.011, *F* = 6.003; day 6 *p* = 0.006, *F* = 6.959; day 7 *p* = 0.004, *F* = 2.797). (B) Area under the curve (AUC), which sums total viral RNA shed, varied significantly by diet (ANOVA *p* = 0.002, *F* = 9.389). Suboptimal-fat diet bats cumulatively shed less than suboptimal-sugar (Tukey-HSD, *p* = 0.001) and standard diet bats (Tukey-HSD, *p* = 0.032). Boxplots show median, 25th and 75th percentiles and range or 1.5 × IQR. Asterisks indicate: *** *p* < 0.0005, ** *p* < 0.005, * *p* < 0.05, **·** *p* < 0.1.

### (c) Diet influences duration and quantity of H18N11 viral RNA shedding

Next, we determined whether there were differences in H18N11-IAV viral RNA (vRNA) shedding between the diet groups. There were no significant differences between the standard and suboptimal-sugar diets on any day, although suboptimal-sugar diet bats trended toward higher vRNA shedding on all days measured (figure 3A). Unexpectedly, bats on the suboptimal-fat diet shed less vRNA on days 4−7. While not significant, the average shedding duration between diets followed the same trend (suboptimal-sugar = 8.3 days, standard = 7.7, suboptimal-fat = 6.7). Bats continued to shed vRNA through day 15, although no individual bat shed continuously.

Using area under the curve (AUC) analysis to quantify total vRNA shed per bat, we found total shedding varied significantly by diet (figure 3B). Standard diet bats trended toward shedding less vRNA than suboptimal-sugar diet bats, but the difference was not significant. Suboptimal-fat diet bats cumulatively shed less vRNA than both suboptimal-sugar and standard diet bats.

### (d) Diet but not infection influences rectal tumor necrosis factor expression

Because we found that diet affected the quantity and duration of vRNA shedding, we next tested whether infection affected antiviral and/or inflammatory responses.

**Figure 4.**
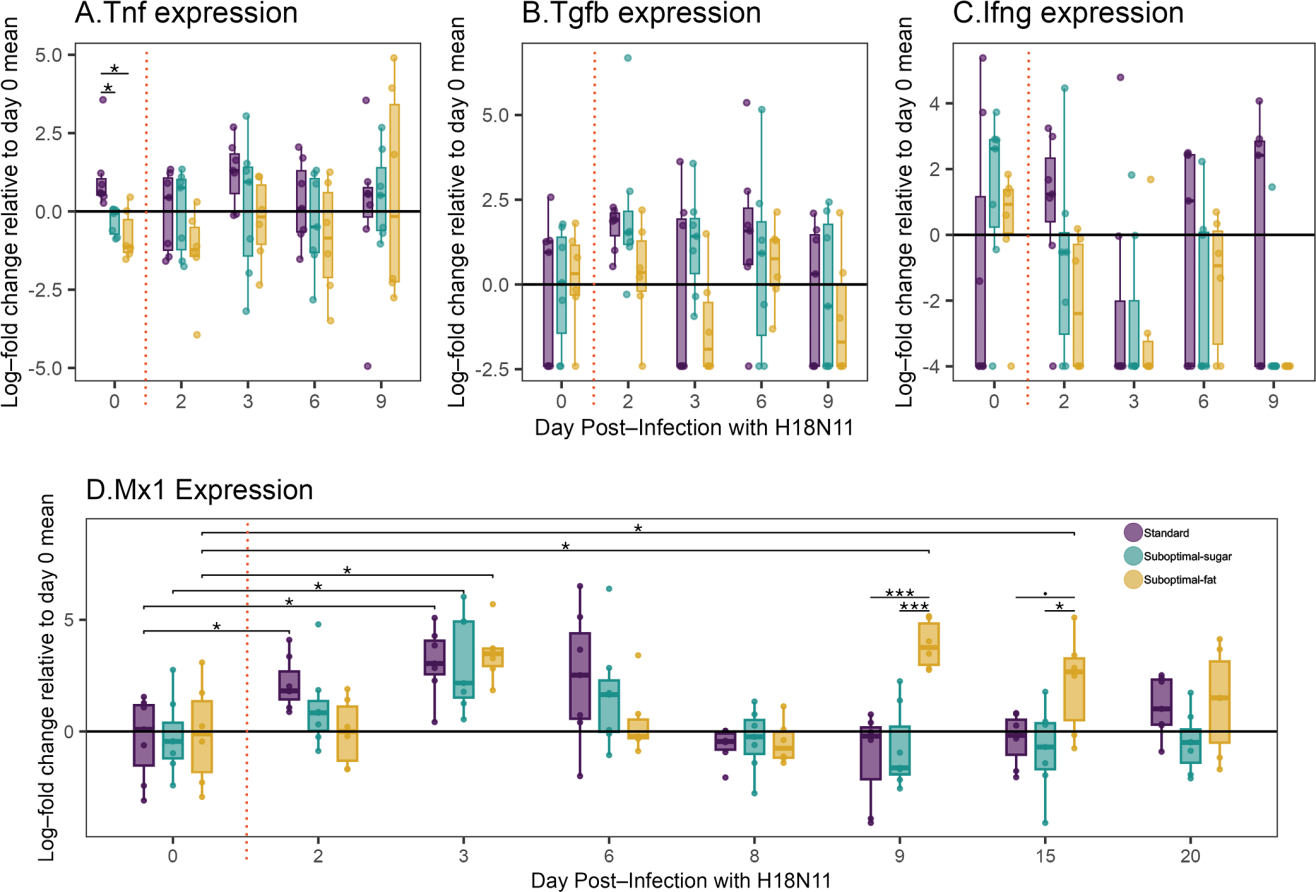
Gene expression from rectal swabs on days 0, 2, 3, 6, 9 (Tnf, Tgfb, Ifng, Mx1) and days 8, 15, 20 (Mx1). Values are natural log-fold change in gene expression relative to day 0 mean expression of all bats. Vertical red dotted lines denote pre- versus post-infection days. (A) *Tnf*: Between-diet differences were significant on day 0 (ANOVA, *F* = 8.692, corrected-*p*‐value = 0.03). (B) *Tgfb* expression. (C) *Ifng* expression. (D) *Mx1*: Between-diet differences: day 9 (ANOVA, *F* = 16.17, *t* = 2, corrected-*p*‐value < 0.001) and day 15 (ANOVA, *F* = 4.99, *t* = 2, corrected-*p*‐value = 0.069). Within-diet differences: compared with day 0 baseline, standard diet bats increased Mx1 expression at days 2 and 3 (paired *t*‐test, day 2 *t* = −2.85, df = 6, corrected-*p*‐value = 0.049; day 3 *t* = −4.17, df = 6, corrected-*p*‐value = 0.029). Suboptimal-sugar diet bats also increased expression at days 2 and 3 (day 2 paired *t*‐test; *t* = −2.17, df = 6, corrected-*p*‐value = 0.105; day 3 *t* = −4.33, df = 6, corrected-*p*‐value = 0.03). Suboptimal-fat diet bats had a delayed response and did not increase Mx1 expression until day 3 (paired *t*‐test: *t* = −3.27, df = 5, corrected-*p*‐value = 0.044). For all diets, Mx1 expression peaked day 3 and returned to baseline at day 8. However, suboptimal-fat diet bats had a second peak of Mx1 at day 9 (paired *t*‐test, *t* = −3.46, df = 5, corrected-*p*‐value = 0.044) and did not return to baseline until day 20 (day 15 paired *t*‐test, *t* = −3.45, df = 5, corrected-*p*‐value = 0.044). Boxplots show median, 25th and 75th percentiles and range or 1.5 × IQR. Asterisks indicate: *** p < 0.0005, ** *p* < 0.005, * *p* < 0.05, **·** *p* < 0.1.

Pre-infection (day 0), tumor necrosis factor (Tnf) expression was highest in standard diet bats and lowest in suboptimal-fat diet bats (figure 4A), suggesting that diet influenced inflammatory state, regardless of infection. Although Tnf expression in standard diet bats was slightly higher than suboptimal diets on day 3, there were no other within-day differences in Tnf expression post-infection. Levels of Tgfb and Ifng were slightly higher in standard diet bats relative to suboptimal diets on several days (figure 4B,C; day 2, 6 Tgfb; day 3 Ifng).

We determined whether cytokine expression was influenced by infection by comparing expression relative to the day 0 baseline. On day 2 post-infection, there were slight changes in expression of Tgfb and Ifng in most bats. Standard diet and suboptimal-sugar bats had small increases in Tgfb (figure 4B). Standard diet bats had a small increase in Ifng while both the suboptimal diets had a small decrease in Ifng (figure 4C). On days 3−9, most bats had undetectable levels of Ifng. Overall, it appears that diet led to differences in baseline expression, but H18N11-IAV infection induced only moderate responses in the cytokines measured.

### (e) Antiviral protein Mx1 had diet-specific expression patterns

Mx1 is an interferon-stimulated antiviral protein important for anti-IAV immune response in humans and mice [41]. Pre-infection Mx1 expression was similar between diets, although all bats were expressing detectable Mx1 (figure 4D).

Relative to baseline (day 0), Mx1 expression increased more rapidly in standard and suboptimal-sugar diet bats (days 2, 3). Suboptimal-fat diet bats had a delayed response and did not increase Mx1 expression until day 3. For all diets, Mx1 expression peaked at day 3 and decreased back to baseline at day 8. However, in suboptimal-fat diet bats, a second peak of Mx1 occurred at day 9 and did not return to baseline levels until day 20 (figure 4D).

### (f) Infection-driven metabolic changes are diet-specific in the gut but not in circulation

**Figure 5.**
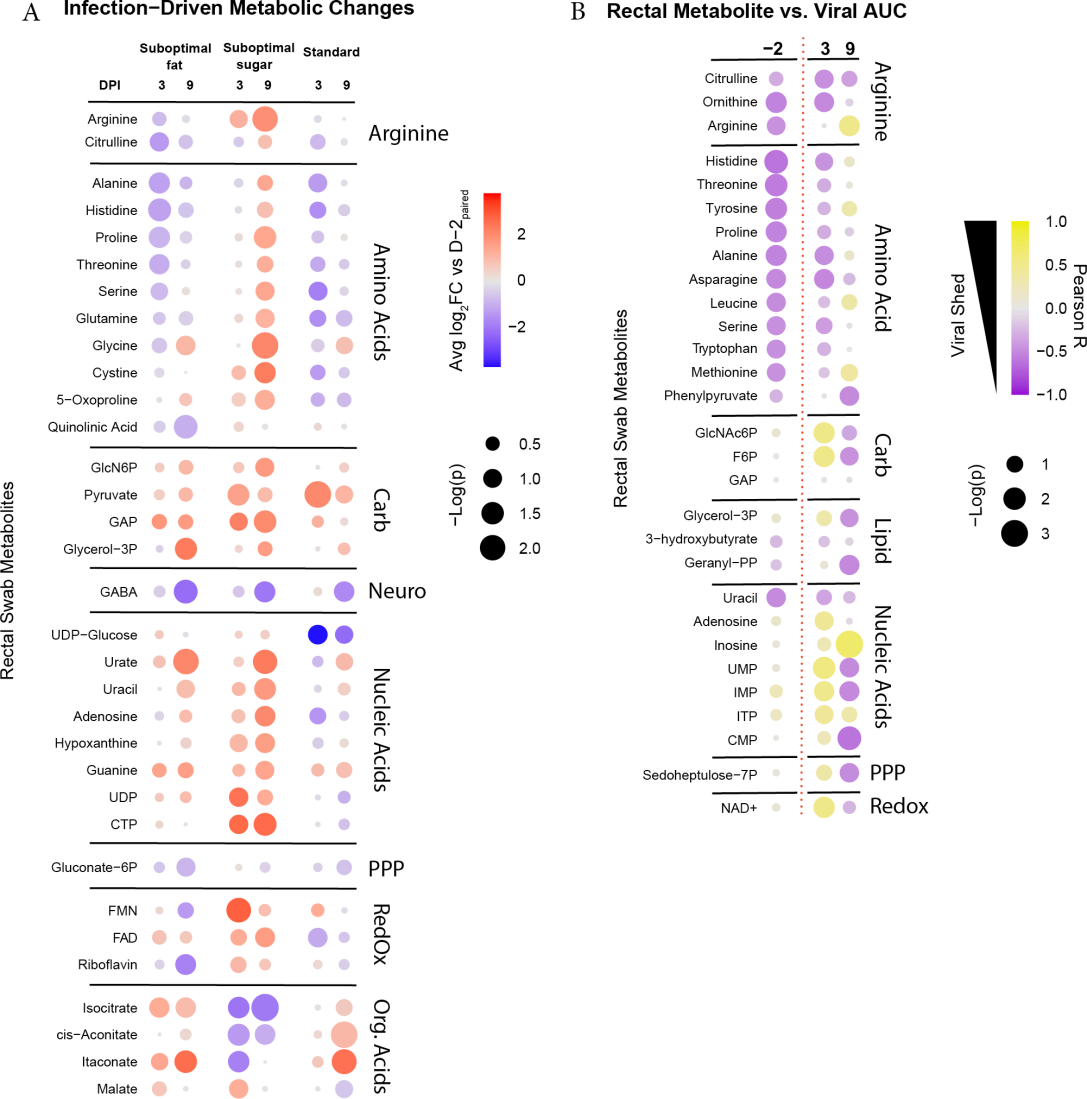
(A) Metabolites in rectal environment that changed relative to pre-infection (day −2) levels on days 3 and 9 post-infection (when bats were still shedding detectable vRNA). Colour indicates log_2_-fold change relative to day −2 values; size of dot indicates –log(*p*). Analysis was performed separately for each diet group owing to differences in metabolite levels pre-infection. Infected bats were also compared with uninfected bats on each day post-infection to adjust for metabolic changes over the span of the experiment. (B) Metabolites from the rectal environment correlated with viral AUC for each bat pre-infection (day −2) and days 3 and 9 post-infection. Diet was not considered in this calculation and each infected bat was treated individually. Colour indicates positive correlation with AUC (i.e. higher levels of metabolite correlated with higher total virus shed) versus negative correlation with AUC. Size of dots indicates –log(*p*). Vertical red dotted line denotes pre- versus post-infection days. DPI, days-post-infection

We looked for correlations between metabolites and viral shedding by determining which metabolic changes were triggered by viral infection in the gut (rectal swabs; figure 5A) or in circulation (plasma; electronic supplementary material, figure S4).

The rectal compartment displayed infection-triggered responses in amino acids, carbohydrate metabolism, nucleic acids, flavin-associated cofactors and TCA-associated organic acids (figure 5A). Few of these responses were conserved across diets, except for the changes in carbohydrate metabolism. The suboptimal-fat and standard diet bats had a decrease in amino acids on day 3. The suboptimal-sugar diet bats had an opposite pattern, with a strong increase in amino acids by day 9. Suboptimal-sugar diet bats also displayed a more robust increase in nucleic acid and nucleic acid metabolites throughout infection. An exception was urate, which showed increases in both suboptimal diets at day 9 post-infection.

In contrast to the gut, post-infection patterns in plasma were largely conserved between diets, but included changes in amino acids, carbohydrate metabolism, nucleic acids and organic acids (electronic supplementary material, figure S4). While the magnitude differed between diets, the directionality and timing were largely preserved.

### (g) Gut levels of amino acids pre- and post-infection are negatively correlated with viral shedding

We next asked which pre-infection metabolite levels and infection-associated metabolic changes had explanatory potential for the differences observed in viral shedding. To this end, we looked for correlations of individual viral AUC with metabolite profiles on day −2 and each day following with both metabolite data and detectable vRNA (days 3, 9; figure 5B, electronic supplementary material, figure S5). Diet was not considered in this calculation so that we could search for common metabolites in all diets that related to total vRNA shed.

Pre-infection levels of amino acids in the gut were strongly negatively correlated with viral AUC (figure 5B). This is consistent with higher rectal levels of amino acids in the lower shedding suboptimal-fat diet bats (figure 2B), but holds across diets (e.g. suboptimal-fruit diet bat with lowest viral AUC had highest pre-infection levels of histidine). This correlation continued until day 3 post-infection, near peak vRNA shedding and was lost by day 9, consistent with the infection-driven increase in amino acids in the suboptimal-sugar diet bats at day 9 (figure 5B). While no pre-infection gut metabolites were positively correlated with viral AUC, day 3 carbohydrate metabolites and nucleic acids positively correlated with viral AUC. Of interest, we found a strong negative correlation between pre-infection levels of arginine cycle members (arginine, citrulline, ornithine) and viral AUC. Pre-infection levels of all three metabolites were higher in suboptimal-fat diet bats (electronic supplementary material, figure S6), suggesting that diet influenced the arginine cycle.

Plasma metabolites were less correlated with viral AUC, consistent with the observed conservation of infection-triggered plasma metabolite patterns between diets (electronic supplementary material, figure S5). Notably, plasma hypoxanthine and xanthine levels both pre-infection and on day 3 were positively correlated with viral AUC, consistent with these metabolites being lowest in the plasma of suboptimal-fat diet bats. This trend was maintained across diets as well (e.g. suboptimal-fat diet bat with highest viral AUC also had highest pre-infection levels of plasma hypoxanthine; suboptimal-fruit diet bat with lowest viral AUC had lowest pre-infection levels of plasma hypoxanthine).

### (h) Suboptimal-fat diet bats have distinct plasma antioxidant response

Because diet led to systemic changes in metabolites associated with redox processes (e.g. xanthine, hypoxanthine, flavin cofactors), we measured enzymatic activity of glutathione peroxidase (GPx) and superoxide dismutase (SOD) in haemolysate samples. Pre-infection, GPx and SOD activity were similar in all diets (electronic supplementary material, figure S7). On day 3 post-infection, suboptimal-fat diet bats had the lowest GPx (electronic supplementary material, figure S7A) and highest SOD (electronic supplementary material, figure S7B). While GPx activity returned to baseline by day 9 in suboptimal-fat diet bats, SOD remained elevated. Despite metabolic signatures of oxidative stress in the rectal metabolome of suboptimal-sugar diet bats and in the plasma metabolome of standard diet bats (i.e. hypoxanthine, xanthine), we observed no differences in these diets.

## 3. Discussion

Bats host many viruses relevant to public health, necessitating research into the immunological and metabolic drivers of viral shedding in wild bat populations. Bats are continuously facing changing landscapes, including loss of native habitat, forcing them to rely on novel food sources. In this study, we attempted to recapitulate these dietary changes in an experimental setting. As hypothesized, Jamaican fruit bats (JFBs) on the suboptimal-sugar diet shed the most H18N11-IAV viral RNA (vRNA). However, contrary to our expectations, bats on the suboptimal-fat diet shed the least vRNA. Bats on suboptimal diets consumed more food, despite only small changes in weight. Furthermore, the suboptimal-fat diet bats had distinct patterns of cytokine, antiviral protein, metabolite and antioxidant enzymes that suggest diet mediated a change in their metabolism and immune response, which influenced vRNA shedding. The duration of pathogen shedding, or infectious period, is important for understanding how pathogens are spread and maintained in populations [42]. Spatial behaviour determines the risk of encounters between bats and secondary hosts (e.g. livestock, humans) and foraging patterns are an important aspect of spatial behaviour. Our results suggest that periods of dietary stress may alter bat behaviour and patterns of pathogen shedding, supporting the hypothesis that diet may mediate the interaction between land-use change and spillover events. This experiment is an important step in understanding the interplay between diet, metabolism and the viral immune response in bats.

We observed potential sickness behaviours in our bats whose appetites were altered by infection. Specifically, bats on the suboptimal-fat diet ate more food than bats on the standard diet, although their weights did not vary substantially. This finding is especially surprisingly given the caloric density of the suboptimal-fat diet, which we estimate to be the highest of all three diets. Although we did not calorically restrict the bats, it is unclear whether bats in anthropogenic landscapes are continuously facing caloric deficiencies or if changes in foraging behaviour mainly lead to nutritional changes. We also observed that the suboptimal-sugar diet bats tended to consume more food than those on the other diets throughout the experiment, although the difference was greater post-infection. This result was not surprising, as previous studies have shown that JFBs given low-protein diets consume more food than those given high-protein diets [43]. Wild *Artibeus* bats consume a wide range of fruits [39,44] but rely on protein supplemented from several sources (pollen, insects, leaves) [45,46]. The suboptimal-fruit diet may thus represent the diet in highly modified areas where bats cannot access supplemental protein (e.g. banana cultivars that don’t produce pollen) [38]. However, we found no evidence that bats naturally seek out additional sources of fat in the wild. Consumption of high-fat fruits (e.g. cocos pal—like the suboptimal-fat diet) [16,37,39] may occur owing to the presence of novel food items and changes in foraging behaviour. Thus, we believe our diets represent standard diets and two putative responses to anthropogenic modification of their habitat. If these trends are similar in wild bats, bats on suboptimal diets may spend more time foraging than bats consuming typical native foods. Notably, black flying foxes increased the number of foraging stops during food shortages [16] and yellow-shouldered bats (*Sturnina hondurensis*) in coffee plantations spent more time foraging than those living in intact forests, possibly owing to the lower availability of food [47]. The risk of viral spillover increases with contact between hosts [48], and more time foraging increases the chance of contact with a secondary host. Future studies are needed to clarify how diet versus caloric restriction and viral infection might alter foraging behaviour. Nonetheless, our results suggest that in addition to the heterogeneity in viral shedding, there may also be a behavioural change that further affects viral transmission dynamics within populations [42].

We identified metabolic markers of diet and viral shedding that relate to gut health, including citrulline [49–51], arginine [49,52] and hypoxanthine [53,54]. The gut metabolite patterns suggest that the suboptimal-fat diet induced a distinct metabolic environment pre-infection that may have primed bats’ immune response, leading to less vRNA shedding and a shorter shedding duration. Based on research in mice and humans, we suspect that higher levels of citrulline and arginine in the gut pre-infection may have reduced inflammatory immunopathology and improved intestinal epithelium healing in response to infection [50,51], allowing for more rapid control of viral infection. Citrulline is locally converted to arginine and then metabolized by nitric oxide synthase to nitric oxide (NO), an important metabolite in immune cells for signaling and anti-viral responses, and in endothelial cells for normal vascular function and gastrointestinal cell integrity [55–57]. Similarly, hypoxanthine improves intestinal barrier function and wound healing [54], which could enhance resistance to intestinal virus infection. Hypoxanthine is also important in energy metabolism and is a precursor for the purine salvage pathway, including generation of adenylates (AMP, ADP, ATP). The pre-infection levels of nucleic acids, nucleobases and amino acids tended to be lowest in suboptimal-sugar diet bats and highest in suboptimal-fat diet bats, suggesting that the suboptimal-fat diet bats had greater resistance to infection and a greater pool of energetic and immune precursors [54,58]. Thus, the diet-driven changes in gut metabolites both pre- and post-infection provide a cohesive potential explanation for the differences in viral shedding. Although the exact mechanism remains unknown, there is recent evidence that the bat microbiome may confer anti-viral resistance [59], suggesting an important potential role of diet-induced changes in the gut microbiome. Future studies should address whether the influence of diet on viral shedding is restricted to gastrointestinal viruses (e.g. H18N11-IAV; coronaviruses), if it can more broadly apply to viruses with different tissue tropism (e.g. henipaviruses) and how metabolic changes across the gut and systemic compartments link diet, microbiome and host antiviral responses.

Suboptimal-fat diet bats had distinct patterns of cytokine expression, antiviral protein expression and plasma antioxidant enzyme activity. Tnf is a pro-inflammatory cytokine produced by innate cells early during viral infections, but we did not detect changes in Tnf in response to infection, consistent with other studies in bats [60]. Pre-infection, rectal Tnf levels were lowest in suboptimal-fat diet bats, suggesting diet-driven variation in gut inflammation. This Tnf expression pattern was consistent with the metabolite signature of reduced inflammation in the suboptimal-fat diet bats. In contrast, we observed a clear induction of the antiviral protein Mx1 in response to infection. The standard and suboptimal-sugar diet bats began expressing Mx1 earlier. Additionally, the suboptimal-fat diet bats had a second peak of Mx1. Levels of Mx1 in the suboptimal-fat diet bats remained elevated throughout the experiment, while the other diets returned to pre-infection baselines. It is unclear what caused the second peak in Mx1, which is generally only expressed in response to type I or III interferons as part of the canonical interferon response [41,61]. Although we did not detect vRNA at these time points, it is possible there were residual pathogen-associated antigens (i.e. PAMPs) that elicited additional expression of Mx1. In addition to their distinct Mx1 response, plasma SOD activity increased in suboptimal-fat diet bats in response to infection, which could increase their capacity to prevent the formation of reactive species, suggesting a role of systemic redox balance. Plasma GPx activity did not increase concurrent with SOD, suggesting a complex response in systemic redox balance that we may not have fully captured. Overall, these distinct differences in the metabolic and immune state in suboptimal-fat diet bats may have contributed to the differences in viral shedding, but additional studies will be needed to clarify the role of local and systemic redox balance.

Our results demonstrate the plausibility that changes in diet owing to habitat degradation are a driver of viral shedding, likely by modifying bats’ metabolic and immune state prior to infection. Although it is not understood how viruses are maintained over time within bat populations, heterogeneity in viral shedding has the potential to prolong outbreaks and contribute to temporal maintenance of pathogens [42]. Our findings that dietary changes influence the duration of viral shedding have implications for how viral transmission dynamics among wild bats are modelled and understood, and they highlight the need to consider ecological factors when studying bat-borne diseases. Our observation that bats on both of the suboptimal diets consumed more food post-infection could translate to increased foraging time in wild bats, increasing the risk of contact with secondary hosts. In wild bats, dietary shifts driven by habitat change may therefore modulate the dynamics of zoonotic pathogens and the risk of spillover to humans.

## 4. Methods

### (a) Bat husbandry and diet

Male Jamaican fruit bats (*Artibeus jamaicensis*; JFBs) were obtained from a specific pathogen-free breeding colony at Colorado State University and acclimated for three weeks. All care and procedures were in accordance with NIH, USDA and the Guide for the Care and Use of Laboratory Animals (National Research Council, 2011). Animal protocols were reviewed and approved by the Montana State University Institutional Animal Care and Use Committee (IACUC 2021−183-IA). Montana State University is accredited by the Association for Assessment and Accreditation of Laboratory Animal Care (AALAC; accreditation no. 713).

Bats were housed on a 12 h on/off light dark cycle at 75+/–2°F (approx. 23.8° C) and 30–70% humidity (IACUC 2021−183). Bats were randomly assigned to diet groups and to infected versus control groups. Throughout the experiment, bats in each cage ate a distinct diet: a standard diet (fruit with protein supplement: Mazuri Exotic Animal Nutrition SKU 0053414), a suboptimal-sugar diet (fruit alone without any supplement), or a suboptimal-fat diet (fruit with a fat supplement: coconut oil). See electronic supplementary material, tables S1 and S2 for nutritional information on each supplement. Each diet is considered within the possible range of food that these bats consume, but the suboptimal-sugar and suboptimal-fat were hypothesized to be less optimal. Bats were fed *ad libitum* since wild bats commonly alter feeding behaviour in response to food availability to avoid starvation [17,62]. Fruit given to each cage was identical except for the supplement, and bats were maintained on this diet for 21 days prior to infection (D−21 to D0) and 20 days post-infection (D0 to D20). Fruits that were provided included banana, apple, orange, cantaloupe and honeydew. JFBs are considered generalists [44,47] and all these fruits are given to them regularly in captivity. Approximately 2 tablespoons (approx. 15 g) of supplement were added to fruit for the cage of bats each day.

To monitor consumption, leftover food was weighed to determine if there were notable differences in food consumed at a cage level. After infection with H18N11, food was weighed both before and after it was given to the bats every day to monitor changes in consumption more precisely. Control bats were housed separately from infected bats. Bats were monitored for 20 days post-infection and then euthanized.

### (b) H18N11 infection

The A/flat-faced bat/Peru/033/2010 (H18N11) virus was rescued by using eight plasmid reverse genetic system as described previously [63,64]. The rescued H18N11 virus was amplified in RIE1495 cells and titrated in MDCK cells [63]. After 21 days on their respective diets, we infected the JFBs oro-nasally with the H18N11 virus. We inoculated each JFB with 5 × 10^5^ TCID_50_ of the H18N11 virus. We randomly selected two JFBs from each diet group to act as controls, inoculating them with equal volumes of sterile saline.

### (c) RNA qRT-PCR: gene expression and viral RNA

Rectal swabs (Puritan 6’ Sterile Mini-tip swabs #25−800−1PD−50) were submerged in DNA/RNA Shield (Zymo R1100) immediately after collection. Rectal swabs were collected on days −21, −15, −8, −2, 0, 2−15 and 20. Nucleic acids were extracted from rectal swabs using Zymo Quick-DNA/RNA Pathogen Miniprep kit following manufacturer’s instructions. Viral RNA was measured using TaqMan Fast Virus 1-Step RT-PCR standard parameters. Primers were designed for the NP region (per [63]) (electronic supplementary material, table S3). Cytokine expression levels were measured using iTaq Universal SYBR Green One-Step Kit (BioRad #172−5151) after being treated with DNAse (Turbo DNA-Free Kit, Ambion #AM1907) using standard PCR conditions (electronic supplementary material, table S3).

We selected these cytokines/anti-viral proteins because we chose a targeted approach focused on anti-viral and immune mediators known to be activated in response to influenza virus infections. Specifically, we selected Tnf as a classic proinflammatory cytokine known to be important in early response to infections in the gastrointestinal tract and Tgfb as a classic regulatory cytokine known for its either synergistic or antagonistic relationship with Tnf. We chose Ifng owing to its relevance to the later stages of anti-viral immune response, and because a recent study in mice found that high-fat diets influenced levels of Ifng and MHCII (major histocompatability complex II) in the intestine [65]. Mx1 was selected because it is an important component of the interferon signaling response (and is also an interferon-stimulated gene), known to confer resistance to influenza virus infections.

### (d) Metabolomics

Plasma (EDTA, Greiner BioOne), rectal swabs (Puritan 5.5’ Sterile Mini-tip Rayon swabs, #25−800-R50) and liver were collected for metabolomics analysis from all bats. Plasma and rectal samples collected during regular sampling were immediately placed into cold methanol then frozen until analysis. Plasma samples were collected on days −21, −15, −8, −2, 3, 9, 15 and 20. Rectal swabs were collected on days −21, −15, −8, −2, 0, 2−15 and 20. Liver sections were collected during necropsy.

For all metabolite extraction methods, derivatization and liquid chromatography-mass spectrometry (LCMS) methods, LCMS grade solvents were used. Tributylamine and all synthetic molecular references were purchased from Millipore Sigma. LCMS-grade water, methanol, isopropanol and acetic acid were purchased through Fisher Scientific.

Plasma samples in methanol were brought to 800 µl with 1 : 1 methanol : water and 400 µl of chloroform were added. Liver samples were bead beaten in 400 µl methanol. Once samples were homogenized, 400 µl of water and 400 µl of chloroform were added. Rectal swabs were collected in 500 µl of methanol to which 500 µl of water and 500 µl of chloroform were added. All samples were shaken for 30 min at 4°C and centrifuged at 16 000xg for 20 min. The top (aqueous) layer was taken for downstream analysis.

Short chain fatty acids (SCFA) were derivatized with O-benzylhydroxylamine (O-BHA) to prevent vaporization using previously established methods [66]. Briefly, to 35 µl of each extracted liver and rectal swab sample were added 10 µl of 1M O-BHA and 10 µl of 1M 1-ethyl−3-(3-dimethylaminopropyl)carbodiimide, both in a reaction buffer consisting of freshly prepared 1M pyridine and 0.5 M hydrochloric acid in water. Samples were shaken at room temperature for 2 h and then quenched via addition of 50 µl 0.1% formic acid. Derivatized carboxylic acids including SCFAs were extracted by adding 400 µl ethyl acetate to each reaction. Following mixing and centrifugation to induce layering, the upper (organic) layer was collected and dried under vacuum. Samples were resuspended in 300 µl of water for LCMS injection.

All samples were separated using a Sciex ExionLC™ AC system and measured using a Sciex 5500 QTRAP® mass spectrometer. Aqueous-soluble metabolites were analysed using an ion pairing method [67]. Quality control samples consisting of a mixture of 10 or more experimental samples from each biological matrix were injected regularly to control for instrument stability. Samples were separated on a Waters™ Atlantis T3 column (100 Å, 3 µm, 3 mm x 100 mm) using a binary gradient from 5 mM tributylamine, 5 mM acetic acid in 2% isopropanol, 5% methanol, 93% water (v/v) to 100% isopropanol over 15 min. Two distinct multiple reaction monitoring (MRM) pairs in negative mode were used to measure each metabolite.

Derivatized SCFA samples were separated on a Waters™ Atlantis dC18 column (100 Å, 3 µm, 3 mm x 100 mm) and eluted using a 6 min gradient of 5–80% B with buffer A as 0.1% formic acid in water and B as 0.1% formic acid in methanol. SCFA and central metabolic carboxylic acids were detected using MRMs from previously established methods and identity was confirmed by comparison with derivatized standards including propionate, valerate, isovalerate, butyrate, isobutyrate and acetate [66,67].

All peaks were picked and integrated using MultiQuant® Software v.3.0.3. Signals were filtered by removing signals with more than 50% missing values and the remaining missing values were replaced with the lowest registered signal value. All datasets were corrected for coherent QC variance via a linear nearest neighbours method. Metabolites with multiple MRMs were quantified with the higher signal to noise signal. Filtered datasets were normalized using the total signal sum prior to analysis. SCFA datasets were stitched to their corresponding polar metabolite dataset via common signals for malate in both methods.

### (e) Antioxidant enzymes

We measured the activity of superoxide dismutase (SOD; Cayman, item no. 706002) and glutathione peroxidase (GPx; Cayman, item no. 703102) in red blood cell lysate following the manufacturer’s instructions. 400 ul of ice-cold Ultrapure water (Cayman item #400000) was added to red blood cell lysate for 1 min, briefly vortexed, centrifuged and supernatant was removed for use in each assay. Total protein in lysate was quantified using a BCA kit (Pierce BCA Protein Assay Kit #23225). SOD and GPx were both standardized to total protein.

### (f) Data analysis

Statistics were done using R v. 4.3.1 (R Core Team 2023−06−16) [68] and the nlme (v. 3.1−162) [69] and mixOmics [70] packages. For metabolomics data, we also used MarkerView® Software 1.3.1 and MetaboAnalyst 5.0 [71].

All data were assessed for normality and either transformed or analysed using non-parametric tests. Unless noted otherwise, diet-specific differences (between-diet comparisons, within a given day) were compared using ANOVAs followed by Tukey-HSD if significant or Kruskal–Wallis followed by pairwise Wilcoxon test if data were not normal based on Shapiro–Wilk test. Between-day differences (within a given diet, looking at changes over time) were tested using paired *t*-tests or Mann–Whitney test if data were not normal based on Shapiro–Wilk test. *P*-values were corrected for multiple tests when multiple tests were run (FDR correction). A PERMANOVA was used to test whether the centroids of the metabolite profiles for diets were different from each other using the vegan package (adonis2; vegan v_2.6−6.1) [72].

Data on weight were assessed using linear mixed effects models (‘lme’, nlme) to determine whether weight changed from the diet (day – 21 to day 0) or owing to infection (day 0 to day 20). Gene expression data were normalized as log fold change relative to pre-infection values of all bats. For calculation of the proportion of food consumed, pre-infection we did not weigh all food prior to giving it to the bats. In order to calculate an estimate of the proportion consumed, we used the average weight of food provided post-infection to the bats. Post-infection, food was weighed before and after so the actual proportion consumed was calculated.

## Data Availability

Data and code are available on Zenodo [73]. Supplementary material is available online [74]
